# Teaching reproducible research for medical students and postgraduate pharmaceutical scientists

**DOI:** 10.1186/s13104-021-05862-8

**Published:** 2021-12-09

**Authors:** Andreas D. Meid

**Affiliations:** Department of Clinical Pharmacology and Pharmacoepidemiology, University of Heidelberg, Im Neuenheimer Feld 410, 69120 Heidelberg, Germany

**Keywords:** Reproducible research, Reproducibility, Heterogeneous treatment effects, Machine learning, Medical education

## Abstract

In medicine and other academic settings, (doctoral) students often work in interdisciplinary teams together with researchers of pharmaceutical sciences, natural sciences in general, or biostatistics. They should be fundamentally taught good research practices, especially in terms of statistical analysis. This includes reproducibility as a central aspect. Acknowledging that even experienced researchers and supervisors might be unfamiliar with necessary aspects of a perfectly reproducible workflow, a lecture series on reproducible research (RR) was developed for young scientists in clinical pharmacology. The pilot series highlighted definitions of RR, reasons for RR, potential merits of RR, and ways to work accordingly. In trying to actually reproduce a published analysis, several practical obstacles arose. In this article, reproduction of a working example is commented to emphasize the manifold facets of RR, to provide possible explanations for difficulties and solutions, and to argue that harmonized curricula for (quantitative) clinical researchers should include RR principles. These experiences should raise awareness among educators and students, supervisors and young scientists. RR working habits are not only beneficial for ourselves or our students, but also for other researchers within an institution, for scientific partners, for the scientific community, and eventually for the public profiting from research findings.

## Introduction

In recent years, there has been a growing awareness that rigorous and transparent reporting of research is needed to ensure that study findings can be reproduced [1]. There is now consensus that the value of research can be enhanced by greater transparency and openness in the processes of research design, conduct, analysis, and reporting [1, 2]. At the same time, however, it became clear that simply registering study protocols, following reporting guidelines alone, or merely providing source data are not sufficient [3, 4]. For this reason, a lecture series on reproducible research (RR) for postgraduate students involved in several areas of clinical pharmacology at our institution has been established. When being offered to give this lecture series, it also appeared to me that even experienced researchers might be unfamiliar with certain aspects of a perfectly reproducible workflow, although reproducibility is of critical importance to any section of our multifaceted and dynamic discipline. This commentary honestly shares useful experiences from the pilot lecture series hoping to encourage reproducible working habits, to emphasize the crucial role of supervisors, and thus strengthen our awareness how important RR is when teaching good research practices. The course was divided into eight lectures, in which the topic was introduced (1), technical requirements were identified (2), R/*RStudio* was presented as a suitable software (3), with which the participants’ own projects (4) could be successively processed according to RR principles. Using pertinent methods for data management (5), data visualization (6), publishing and reporting (7), these projects could be finalized in a reproducible workflow (8). The commentary follows this structure and highlights important findings along the way.

## Main text

### Introducing reproducible research

In the first lecture of the course, RR was introduced by addressing the key questions about what it is by definition, why we should work accordingly, by which means we can conduct reproducible analyses, and how we can profit from them. The very first finding from the course was indeed that different researchers use the terms “reproducible” and “replicable” differently and sometimes interchangeably [5]. Following the “new lexicon for research reproducibility” [6], (methods) reproducibility is based on the same research conditions and is a fundamental requirement for successful replication (results reproduction). Full replication instead involves independent investigators, independent data, and optionally independent methods [7]. If original study results can be reproduced at all (which is rarely enough the case), then an application of the analysis procedure to new data (replication) is all the more meaningful. That such replication studies are very rare [8] shows the current difficulties of an open science philosophy. For example, DeBlanc and co-workers found limited availability of source code to analyse Medicare data in general medical journals [9]. While the authors meticulously explored potential reasons, they also emphasize that this clearly impedes RR. With data and source code at hand, however, all options are possible and allow all opportunities.

There are indeed positive examples for well-reproduced analyses and their added value in the current literature. In particular, in their re-analysis pursuing inferential reproducibility of the PACE (“paracetamol for acute low back pain”) trial data, Schreijenberg et al. were able to confirm the results from earlier analyses of the same data [10]. This re-analysis demonstrated that trial conclusions were reproducible even after replication with a different methodological approach.

To keep attention high during the course, a cautionary negative example was also given with the “Duke-scandal” eventually leading to termination of clinical trials and lawsuits after article retraction [11]. With these drastic consequences in mind, the advantages of RR could be quickly worked out, either for the single researcher (e.g., streamlined working habits, strengthened confidence, easier adaptations), for the research team (e.g., higher research impact), or for the (scientific) community (e.g., better public and inter-professional recognition).

At this early stage of the lecture series, a working example was introduced providing a published analysis to be reproduced. This illustrative example was followed throughout the course. The particular publication of Duan et al. [12] headed into a current direction with high prospects for personalized medicine, namely predicting individual benefit by exploring heterogeneous treatment effects in the SPRINT [13] and ACCORD trials [14]. Based on the available baseline information, the authors developed models predicting individual treatment response to intensive antihypertensive treatment so that (better) treatment decisions could be made. Interestingly, a machine learning approach called *X*-learner [15] outperformed several alternative methods including the conventional logistic regression with interaction terms. In the publication, a publicly available Github repository was cited where the project is shared [12].

### Requirements for RR

In the second lecture, workflow systems were approached to explicitly reflect the structure of projects, automate recurring steps, and transparently record the origin (‘provenance’) of (intermediate) results. Specifically, two possible solutions were assessed, namely the use of so-called visual workflows [16] and clearly defined folder structures. The latter applied to our working example [12], which was well-structured and was generally a very positive example bypassing several known barriers to proper reproducibility. Among the possible barriers are poor standardization of model building, lacking or insufficient documentation, incomplete transparency, or coding and typing errors. These common difficulties can be addressed by following five best practices in statistical computing [17], namely (1) best practices in code writing and commenting that also (2) documents workflow and key analytic decisions, (3) careful version control, (4) good data management, and (5) regular testing and review. All these aspects are easy to implement and understand from a standardized folder structure. Following our positive working example, Table 1 shows an exemplary pattern of how a research project could be structured. Centrally located in the main folder should be a readme-file in which important project information is documented. This includes, for example, the software packages with the corresponding version. Mainly, however, this concerns which files have to be executed in which order to get intermediate and final results. The main project folder should also include a file with which the entire project can be analysed. In this (make-) file separate analysis scripts are executed (i.e., “sourced”), which run the necessary code lines for data preparation, statistical modelling or result generation (here collected in the subfolder “src”). (Raw) data are read in from a corresponding subfolder. If cross-project or recurring functions have to be used for the analysis, they can be stored in a library folder (here called subfolder “lib”) (as well as other documents). Intermediate and final results can be stored automatically in a separate subfolder. For the publication and dissemination of the results, a separate subfolder is useful, which can be used for technical reports, presentations, manuscripts or even interactive apps. The Github repository of our working example was created accordingly [12].

**Table 1:** Sample principle of a workflow for RR with a standardized folder structure

Main folder	Contents	Comment on item
Project name		Naming according to recurring pattern, which may include initials of the researcher and the date
└	Readme	Mandatory (text) file with important project information on prerequisites, scientific and technical background, and an instruction how to run the project code. Can also include a list of necessary software (package) versions, if not supplied as a separate file
└	Folder “data”	Folder with (raw) data or preprocessed data
└	Folder “lib”	Folder for storing literature or cross-project scripts (e.g., R functions, R packages, …)
└	Folder “results”	Folder for saving results of any kind (tables, figures, R-images, …)
└	Folder “src”	Folder with all executable [“source()”] files
└	Folder “paper”	Folder for storing publication drafts of any kind (e.g., Word documents, Markdown results, Shiny apps, …)
└	Folder “old”	Optional collection folder for old version of scripts or similar
└	Make	Central executable files for reproduction of the project

### Practical reproduction of a working example

From the third lecture on, the basics in using the software R were established. In addition to general techniques for data management and plotting, more specific skills were taught, as they may be necessary for RR. Thus, key concepts and their technical solutions were introduced (e.g., literate programming with R-markdown), and the advantages of common standards were emphasized. All course participants were able to bring in their own projects, which were worked on during the course. Inspired by the advantageous starting position of our working example, the published findings should now be reproduced, as well. Due to the fact that all lecture contents were now practically carried out on the working example in parallel, this working example accompanied the course until the end.

The Github repository of our working example provided the source code written in the Python programming language [12]. Before the code could be run, the data repository storing the source data required that ethical committee approval had to be obtained before data access could be granted [18]. After that, a workspace environment for the Python programming language had to be set up. This did not appear straightforward, but an intuitive readme file was helpful together with other ‘best practice’ ideas, standardized folders and files to be sourced for automatic data loading and running of the analysis code. The repository in particular provided several conceptual benefits when it comes to making distinctions between source data and derived files. It also helped to recognize the dependencies between code elements, different files, or libraries. In the end, the project could indeed be run and yielded results in the identical fashion to the published paper.

**Fig. 1:**
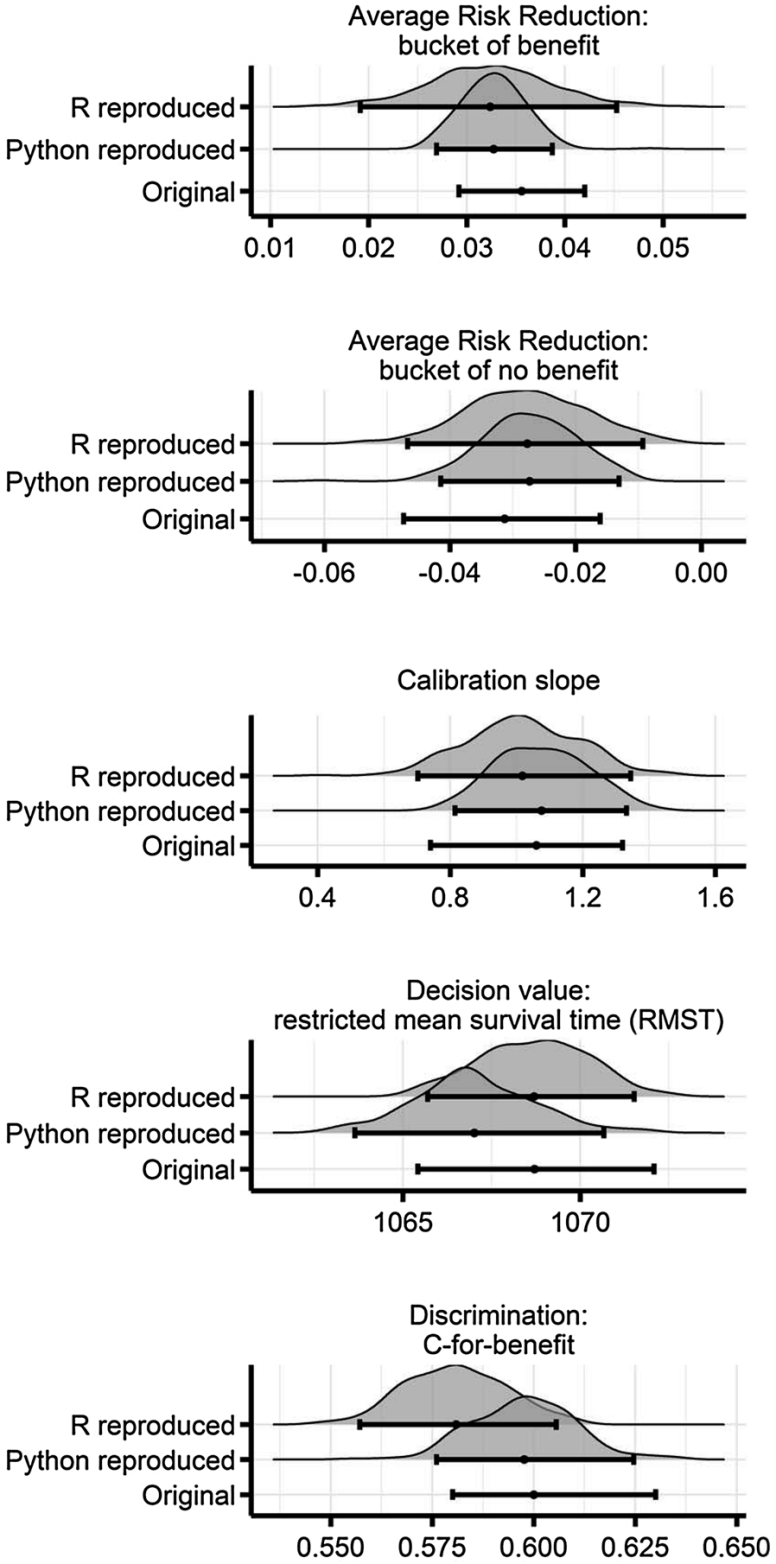
Bootstrapped performance metrics used to derive mean estimates and 95% confidence intervals from the original publication [12], from the reproduction using the supplied code written in Python, and from the supplied code translated to R. Average risk reductions were calculated for two subgroups (buckets) of those patients with predicted benefit in absolute risk reduction (ARR  > 0) and those patients without predicted benefit (ARR  ≤ 0). A calibration line was fitted between quintiles of ARRs and predicted risk, whose slope is chosen for this set of performance metrics. As a decision value, the model predicted restricted mean survival time [RMST (days)] indicates the mean time to event if treatment choice would have been based on the predicted individual benefit [and is thus to be compared with the baseline value of 1061.2 days, 95% confidence interval: (1057.4; 1064.1)]. The c-for-benefit is a metric reflecting the model’s ability to predict treatment benefit (rather than risk for an outcome) [20]. Using the Python implementation to calculate this metric, the individual risk estimates reproduced in R yielded an estimate of 0.61 (0.55; 0.72). Of note, we restrict the presentation of results to distributions from resampling and their summary parameters; further numerical metrics to quantify reproducibility are left out for simplicity. Analyses were using the Anaconda distribution of Python version 3.7.3 (Anaconda Software Distribution, version 2–2.4.0) and the R software environment version 3.6.1 (R Foundation for Statistical Computing, Vienna, Austria)

The published analysis apparently satisfied all three levels of reproducibility, full depth (i.e., code for data preparation, analytical results, and figures), portability to another computer system, and full coverage (i.e., all published results) [19]. Nevertheless, slightly diverging results and the fact that Python module versions have changed since the original publication were the reason to translate the Python code to more familiar R code as a more common programming language in medical research. Any colleague being not familiar with dedicated analysis software could have tried to reproduce the findings by this means. Interestingly, resulting estimates from this particular attempt to reproduce the original analyses were even more intriguing. Figure 1 illustrates several metrics from the original publication describing the performance of the *X*-learner in predicting individual treatment benefits to intensified blood pressure control.

All performance metrics relied on bootstrapped samples to derive estimates for their mean and 95% confidence intervals. For the most part, the Python and R results clearly overlapped suggesting good agreement and thus reproducibility (or “inferential” reproduction when considering that two different analytical methods were actually used). Nevertheless, there were also relatively large differences, especially in the absolute risk reduction in the group (“bucket”) with predicted benefit of intensified blood pressure reduction or the c-for-benefit metric [20] with significant shifts in the distribution of bootstrap estimates. If concrete conclusions in terms of treatment recommendations are drawn from this, these differences could become relevant. A closer look yet revealed more aspects that are likely to explain the differences, especially between the two analysis software packages. One possible explanation is that Duan and co-workers used inverse probability of censorship weighting [21] to handle the time-to-event nature of the source data. For predicting the (survival) probability of no censorship, different external functions were loaded into Python and R that produced slightly different probabilities. But also custom generic code can be an issue upon translation. In order to calculate the c-for-benefit [20] in R, the R code provided in the supplements of the original publication was used and yielded a slightly smaller estimate. Among general explanations, the fact that random forests are indeed based on a random process is the most compelling argument, especially when they have to be reproduced with different programs on different operating systems (and thus different seeds) [22].

### Lessons learned and implications

In the final lecture with project presentations, the lessons learned from the attempts to reproduce the working example were discussed. These experiences from the lecture series’ working example expose several noteworthy aspects. Good documentation, standardized workflows, available data, and a freely available software solution facilitate RR. In this particular case, this framework likewise helped to teach and better understand cutting-edge methods. By deciding to reproduce this excellent work, a prototypical positive example was provided that simultaneously elucidated barriers that any of us could have trying to reproduce these findings. Considering the potential benefits of RR and our surprising observations and obstacles, the key question is how to incorporate these insights into typical workflows of medical research.

Obviously, data availability is a central aspect for reproducibility. Potential barriers might relate to data ownership, data privacy, or information enabling to identify patients. While considering the risk for misuse, protections should not preclude the incredible potential of accessible data for research. There are several pertinent solutions ranging from public or private archives (enabling full data access) to public or private enclaves (allowing to obtain only aggregated results according to specific queries) [23].

Commonly available software tools and a common analysis language are further decisive points facilitating reproducible working habits. The endeavors of the pharmacometric community towards standards in Modeling and Simulation are interesting to follow [24]. Here, repositories sometimes include simulated data sets in accordance with actual distributions and correlations as a pragmatic solution to data privacy and security requirements. Pseudo-code can also be helpful, although a description alone will mostly not be sufficient for RR [4]. If pseudo-code is used, it should be as precise as possible, because the devil is in the details [25]. This makes the idea of a common analysis language across certain software packages seem all the more appealing, as it was approached in the field of Pharmacometrics [26]. As inter-operability of programming languages is more and more common, sharing our research projects in accordance with RR principle would be a helpful step forward to facilitated reproduction and hopefully successful replication with independent data in the end.

## Outlook

Hands-on education and scientific interaction stand on top of all requirements. In addition to publications, tutorials, or courses, the principles of RR should also appear in harmonized curricula for quantitative medical researchers. While a clear roadmap for instructors has yet to be defined, a course in RR should consider the following insights from our pilot lecture series:

- As our pilot lecture series profited from different perspectives from inter-professional teams, either the audience or the lecturers should come from different disciplines to better recognize the aims, means, and potential merits of RR.
- As our hands-on experiences from a fully reproduced analysis illustrated good practices and practical obstacles of RR, such a course should be oriented towards practical examples.
- As scientific interactions were very enriching, a course of RR should include enough room for discussions.
- As skills in statistical programming are fundamental to apply best practices, such a course should provide the basics or be accompanied by another course.

If these efforts further supported reproducible projects, it would not only build confidence and credibility within our broad discipline, but also towards other disciplines and decision-makers. In order for our findings to impact regulatory decisions or patient care, results must be replicable in independent settings as the ultimate standard, for which reproducible projects are fundamental [7].

## Data Availability

Access to the underlying data of the published analysis [12] being reproduced for teaching purposes can be requested at https://biolincc.nhlbi.nih.gov/home. As specified in published analysis [12], the corresponding analysis code is available from the repository https://github.com/tonyduan/hte-prediction-rcts. The mapped project folder with translated R code is available from the repository https://github.com/andreasmeid/Duan_reproduction.

## Abbreviations

ACCORD: Action to control cardiovascular risk in diabetes
PACE: “Paracetamol for acute low back pain” trial
RR: Reproducible research
SRPINT: Systolic blood pressure intervention trial
